# A phase 1 study of the safety, tolerability, pharmacokinetics, and pharmacodynamics of TAK-063, a selective PDE10A inhibitor

**DOI:** 10.1007/s00213-016-4412-9

**Published:** 2016-08-30

**Authors:** Max Tsai, Lambros Chrones, Jinhui Xie, Hakop Gevorkyan, Thomas A. Macek

**Affiliations:** 1 Takeda Development Center Americas, Inc., One Takeda Parkway, Deerfield, IL 60015 USA; 2 California Clinical Trials Medical Group, Glendale, CA USA

**Keywords:** Schizophrenia, Pharmacokinetics, Safety, Phosphodiesterase 10A, Single-rising dose, Oral

## Abstract

**Rationale:**

Schizophrenia is a complex neuropsychiatric disorder characterized, in part, by impaired dopamine signaling. TAK-063 is a selective inhibitor of phosphodiesterase 10A, a key regulator of intracellular signaling pathways that is highly expressed in the striatum.

**Objective:**

Safety, tolerability, and pharmacokinetics of TAK-063 were evaluated in a phase 1 study.

**Methods:**

Healthy Japanese and non-Japanese volunteers were randomized into dose cohorts of 3, 10, 30, 100, 300, and 1000 mg. Each fasting volunteer randomly received a single dose of TAK-063 or placebo. Individuals from the 100-mg cohort also received a post-washout, 100-mg dose under fed conditions. A total of 84 volunteers enrolled (14 per cohort).

**Results:**

The most common drug-related adverse events (AEs) were somnolence (33.3 %), orthostatic tachycardia (19.7 %), and orthostatic hypotension (9.1 %). The three severe AEs recorded occurred at the highest doses: orthostatic hypotension (*n* = 1; 300 mg) and somnolence (*n* = 2; 1000 mg). There were no deaths, serious AEs, or discontinuations due to AEs. TAK-063 exposure increased in a dose-dependent manner. Median *T*_max_ was reached 3 to 4 h postdose. Fed conditions slowed absorption (*T*_max =_ 6 h) and increased oral bioavailability. Renal elimination was negligible. Safety and pharmacokinetic parameters were similar between Japanese and non-Japanese subjects. Impairments in cognitive function consistent with the effects of other sedative or hypnotic agents were detected using a validated, computerized cognition battery, CNS Vital Signs.

**Conclusions:**

TAK-063 was safe and well tolerated at doses up to 1000 mg and demonstrated a pharmacokinetic profile supporting once-daily dosing. Further evaluation of the clinical safety and efficacy of TAK-063 is warranted.

**Electronic supplementary material:**

The online version of this article (doi:10.1007/s00213-016-4412-9) contains supplementary material, which is available to authorized users.

## Introduction

Schizophrenia is a neuropsychiatric disorder of complex etiology. Associated with lifelong disability and a poor quality of life, schizophrenia presents as a triad of positive, negative, and cognitive symptoms (Citrome 2014; de Araujo et al. 2012).

Current standard of care (first- and second-generation antipsychotics) are thought to achieve their therapeutic effects primarily through antagonism of the dopamine D_2_ receptor subtype (Farde et al. 1992; Ginovart and Kapur 2012). Numerous studies have demonstrated the efficacy of antipsychotics in ameliorating positive symptoms (Lieberman et al. 2005), though a significant proportion of patients experience residual symptoms that are not responsive to therapy (Lehman et al. 2004; Tarrier 1987). Further, these drugs have little to modest clinical effects on cognitive and negative symptoms (Citrome 2014; Lehman et al. 2004; Leucht et al. 2009).

Current antipsychotics are often associated with a multitude of adverse events (AEs), including extrapyramidal symptoms (EPS) and hyperprolactinemia, which are due, at least in part, to excessive dopamine D_2_ receptor antagonism in the striatum and the pituitary gland, respectively (Farde et al. 1992; Ginovart and Kapur 2012). Metabolic disturbances affecting weight, lipid profile, and blood glucose levels have also been observed following treatment with current agents or therapies (Ginovart and Kapur 2012; Leucht et al. 2009; Newcomer 2007). These dose-limiting side effects, along with the persistent cognitive, negative, and psychotic symptoms also associated with typical and atypical antipsychotics, underscore the need for new pharmacologic treatment options (Citrome 2014).

TAK-063 is a potent and selective phosphodiesterase 10A (PDE10A) inhibitor in clinical development for the treatment of schizophrenia (Suzuki et al. 2015). PDE10A is an intracellular enzyme that degrades cyclic adenosine monophosphate pathway (cAMP) and cyclic guanosine monophosphate pathway (cGMP)—second messenger molecules with important effects on neuronal excitability (Fujishige et al. 1999; Song et al. 2006; Threlfell et al. 2009). Unlike other phosphodiesterases, PDE10A is selectively expressed in the GABAergic medium-spiny neurons (MSNs), the predominant neuronal population of the striatum (Kreitzer 2009; Sano et al. 2008; Seeger et al. 2003; Suzuki et al. 2015).

In MSNs of both the direct and indirect pathways, PDE10A mediates signaling activity downstream of dopamine D_1_ (direct) and D_2_ (indirect) receptor activation (Sano et al. 2008; Siuciak et al. 2006; Suzuki et al. 2015). PDE10A-mediated modulation of signaling through these two distinct dopamine receptor subtypes affects the sensitivity of MSNs to glutamatergic inputs, and thereby partly regulates the opposing effects of both the direct and indirect pathways (Siuciak et al. 2006; Surmeier et al. 2007). PDE10A may represent an important regulatory locus within the striatal signaling network; it receives inputs from cortical, thalamic, and subcortical areas that regulate sensory processing, motor planning, and goal-directed behavior (Alexander et al. 1986; Macpherson et al. 2014; Perez-Costas et al. 2010). Notably, the clinical expression of schizophrenia includes significant deficits in these and related higher-order processes, making it difficult for patients to perform basic tasks (Citrome 2014; de Araujo et al. 2012).

Taken together, the preclinical data validate PDE10A as a potential target for therapeutic intervention (Grauer et al. 2009; Kehler and Nielsen 2011), and support further investigation of the safety, tolerability, and potential multidimensional efficacy of selective PDE10A inhibitors in people with schizophrenia (Heckman et al. 2016). Here, the results of a phase 1, randomized, placebo-controlled, single-rising dose study exploring the pharmacokinetic (PK), and pharmacodynamic (PD) profiles of TAK-063 in healthy subjects are reported. As prior reports have noted ethnic differences in pharmacokinetics of several compounds, results for Japanese and non-Japanese subjects are reported (Fukunaga et al. 2011).

## Methods

### Study design and subjects

The study was conducted at a single site in the USA in compliance with Institutional Review Board regulations, Good Clinical Practice regulations and guidelines, and all local regulations.

Healthy Japanese and non-Japanese subjects between 18 and 55 years of age were recruited to participate in the study. Criteria for exclusion included prior treatment with any investigational compound within 30 days of the first TAK-063 dose, a positive test for drugs of abuse or nicotine, and an increased risk for suicide. Subjects were randomized into 1 of 6 dose cohorts (3, 10, 30, 100, 300, and 1000 mg), with each cohort containing 11 TAK-063 subjects (five Japanese, six non-Japanese) and three placebo subjects (one Japanese, two non-Japanese) receiving a single dose under fasting conditions. Following a washout period of 7 days, subjects in cohort 3 (100 mg) received the same dose (100 mg) but under fed conditions.

### Study endpoints

The study assessed safety and tolerability in all subjects for the 4-day study duration. The primary endpoints were the percentage of subjects who experienced at least one treatment-emergent adverse event (TEAE), as well as the percentage of subjects who met predefined criteria for safety laboratory tests, vital sign measurements, and safety parameters following TAK-063 administration. The secondary endpoints were PK parameters for TAK-063 and M-I, assessing drug exposure at each dose, and the effect of food coadministration on TAK-063 PK. Exploratory endpoints included the effects of TAK-063 on domains of cognition as assessed by a computerized battery, CNS Vital Signs (CNSVS).

### Safety assessments

Adverse events, clinical laboratory tests, vital signs, 12-lead electrocardiograms (ECGs), and Columbia-Suicide Severity Rating Scale (C-SSRS) were all monitored. Intensity of AEs was classified as mild, moderate, or severe, and each AE was classified as to its relation to study drug.

Laboratory tests evaluated hematology, serum chemistry, urine, and hormone levels. Vital signs and ECGs were collected at screening, check-in, within 1 h predose, serially postdose, and at study exit, while the C-SSRS was administered at screening, check-in, and study exit. Any events reported on the C-SSRS and clinically significant laboratory abnormalities were reported as AEs.

### Pharmacokinetic and pharmacodynamic assessments

Serial plasma and urine samples were collected from all subjects prior to dosing and at specific time points or intervals up to 96 h postdose. Pharmacokinetic samples were processed immediately and frozen at −20 °C as duplicate sets. Plasma concentrations of TAK-063 and its metabolite M-I were subsequently measured by validated liquid chromatography-tandem mass spectrometry with a validated range of 0.5 to 1000 ng/mL for both analytes.

The PK parameters analyzed for TAK-063 and its metabolite M-I in plasma and urine included area under the plasma concentration-time curve from time 0 to infinity (*AUC*_(0-inf)_), maximum observed plasma concentration (*C*_max_), time to reach *C*_max_ (*T*_max_), terminal elimination half-life (*T*_1/2_), oral clearance (CL/F), volume of distribution (V_z_/F), and renal clearance (CL_r_). Metabolite-to-parent ratios were estimated from *C*_max_ and *AUC*_(0-inf)_ data.

Cognitive flexibility, attention, executive function, memory, reasoning, motor control, psychomotor speed, reaction time, and information processing speed were assessed predose and at 2 and 6 h following TAK-063 treatment using the CNSVS computerized cognition battery. The CNSVS cognition battery is a well-validated, sensitive measure and included the following tests: verbal and visual memory (VBM and VIM) tests, finger-tapping test, symbol digit coding test, Stroop test, shifting attention test, the nonverbal reasoning test, and the 4-part continuous performance test (Gualtieri and Johnson 2006). Standardized domain scores for cognitive flexibility, composite memory, executive functioning, processing speed, psychomotor speed, reaction time, reasoning, sustained attention, verbal memory, visual memory, and working memory were reported. In all domains, a decrease in the standardized domain score is indicative of impairment.

### Statistical analysis

All statistical analyses were generated using SAS Version 9.2 (SAS, Cary, NC, USA). The PK parameters were derived using non-compartmental methods with WinNonlin Enterprise Version 6.3 (Pharsight Corp., Mountain View, CA, USA). Frequency or descriptive statistics were used for summary of data. Where indicated, percent coefficient of variation was included in the summary of continuous data. All statistical tests were 2-tailed at *α* = 0.05 level for significance unless otherwise stated. A power model was used to evaluate dose proportionality by race for fasted subjects; in the event of no observed race effect, dose proportionality was assessed for all subjects. To evaluate the food effect, a paired *t* test was performed on *T*_max_ and natural log-transformed *C*_max,_ and AUCs. An ANCOVA model with baseline scores and race as covariates was used to assess the effects of TAK-063 on CNSVS domain scores.

## Results

### Demographics

A total of 84 subjects were enrolled in this study, with comparable distribution of male and female subjects in each dose group (Table 1). The mean age and body mass index were similar among all groups and ranged from 30.5 to 36.1 years and 22.6 to 24.5 kg/m^2^, respectively. Over 90 % of subjects were of non-Hispanic/non-Latino ethnicity, and the racial distribution across subjects was approximately half Asian, one-quarter Caucasian, and one-quarter Black/African American.

**Table 1:** Demographics and baseline characteristics of all enrolled subjects

	Placebo (*n* = 18)	TAK-063 3 mg (*n* = 11)	TAK-063 10 mg (*n* = 11)	TAK-063 30 mg (*n* = 11)	TAK-063 100 mg (*n* = 11)	TAK-063 300 mg (*n* = 11)	TAK-063 1000 mg (*n* = 11)	TAK-063 Total (*n* = 66)	Overall (*N* = 84)
Age^a^, mean (SD), years	36.1 (11.13)	32.3 (10.04)	35.6 (9.63)	33.0 (6.24)	30.5 (10.18)	30.5 (12.31)	33.1 (8.23)	32.5 (9.4)	33.3 (9.84)
Sex, %									
Female	44.4	54.5	36.4	45.5	54.5	54.5	54.5	50.0	48.8
Ethnicity^b^, %									
Hispanic or Latino	5.6	18.2	0	9.1	0	18.2	9.1	9.1	8.3
Race, %									
Asian	33.3	54.5	45.5	54.5	45.5	45.5	45.5	48.5	45.2
Black or African American	38.9	0	18.2	27.3	36.4	36.4	18.2	22.7	26.2
White	22.2	45.5	36.4	18.2	18.2	18.2	36.4	28.8	27.4
Multiracial	5.6	0	0	0	0	0	0	0	1.2
BMI^c^, mean (SD), kg/m^2^	24.2 (3.052)	22.6 (2.109)	24.6 (4.205)	23.3 (2.617)	23.8 (2.269)	24.5 (3.581)	24.0 (3.681)	23.8 (3.13)	23.9 (3.101)

*BM* *I* body mass index; *SD* standard deviation

^a^Age at screening

^b^Ethnicity was classified as Hispanic or Latino or non-Hispanic or non-Latino

^c^BMI at baseline, which is defined as the measurement immediately before the first dose of study drug

### Safety

No deaths or other serious AEs were reported, and no subject discontinued treatment because of AEs. Across all cohorts, 32 of 66 subjects experienced a total of 57 drug-related AEs (Table 2). The most common drug-related AEs following TAK-063 treatment were somnolence (33.3 %), orthostatic tachycardia (19.7 %), and orthostatic hypotension (10.6 %). Reports of EPS or EPS-like events were infrequent across all subjects—one Japanese subject reported muscle tightness in the 30-mg treatment group. The majority of AEs (55/63; 87.3 %) were of mild intensity. Three adverse events were reported as severe intensity: one subject had orthostatic hypotension (300 mg), and two had somnolence (1000 mg). The total incidence of TEAEs within Japanese subjects treated with TAK-063 was 43.3 % compared to 52.8 % in non-Japanese subjects (Online Resources 1 and 2). None of the physical examination parameters, clinical laboratory tests, C-SSRS findings, or ECGs was considered clinically significant for any subject. There was an increased incidence of abnormal readings for blood pressure and pulse rate, mostly occurring in subjects in the 1000-mg TAK-063 treatment group.

**Table 2:** Treatment-emergent adverse events across all subjects by dosing group

Adverse event, *n* (%)	Placebo (*n* = 18)	TAK-063 3 mg (*n* = 11)	TAK-063 10 mg (*n* = 11)	TAK-063 30 mg (*n* = 11)	TAK-063 100 mg (*n* = 11)	TAK-063 300 mg (*n* = 11)	TAK-063 1000 mg (*n* = 11)	TAK-063 Total (*n* = 66)
Somnolence	0	0	0	4 (36.4)	7 (63.6)	5 (45.5)	6 (54.5)	22 (33.3)
Orthostatic tachycardia	1 (5.6)	2 (18.2)	0	1 (9.1)	2 (18.2)	1 (9.1)	7 (63.6)	13 (19.7)
Orthostatic hypotension	3 (16.7)	0	0	0	1 (9.1)	1 (9.1)	5 (45.5)	7 (10.6)
Vomiting	0	0	0	0	1 (9.1)	1 (9.1)	1 (9.1)	3 (4.5)
Nausea	0	0	0	2 (18.2)	0	0	1 (9.1)	3 (4.5)
Dizziness	0	0	0	1 (9.1)	0	1 (9.1)	1 (9.1)	3 (4.5)
Dysarthria	0	0	0	0	0	0	1 (9.1)	1(1.5)
Headache	2 (11.1)	0	0	0	1 (9.1)	1 (9.1)	0	2 (3.0)
Anxiety	0	0	0	0	0	0	1 (9.1)	1 (1.5)
Hypotension	0	0	0	0	1 (9.1)	0	0	1 (1.5)
Blurred vision	0	0	0	0	0	0	1 (9.1)	1 (1.5)
Epistaxis	0	0	0	1 (9.1)	0	0	0	1 (1.5)
Muscle tightness	0	0	0	1 (9.1)	0	0	0	1 (1.5)

### Pharmacokinetic properties of TAK-063

**Fig. 1:**
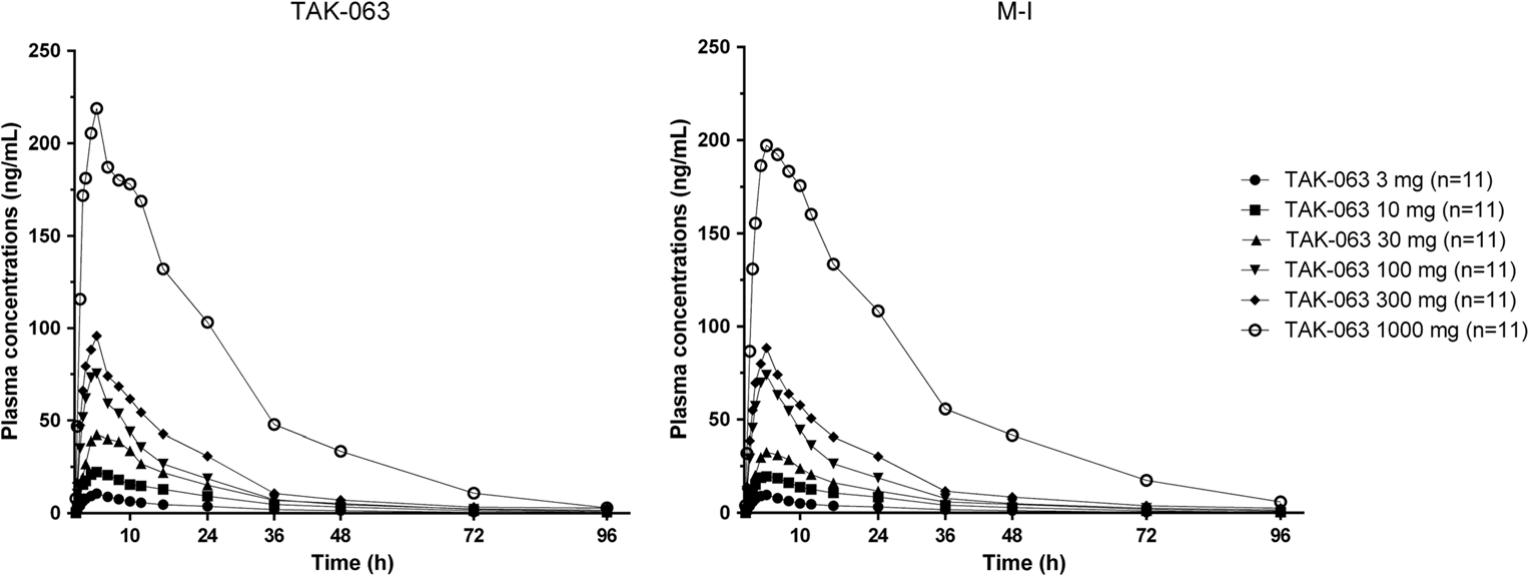
Mean plasma concentration-time profiles of TAK-063 and M-I (metabolite) across all subjects by dosing group

The mean concentration-time profiles of TAK-063 and M-I in all subjects were measured over a period of 96 h following administration of single oral doses (Fig. 1). Under fasting conditions, TAK-063 was absorbed with a median *T*_max_ value of 3 to 4 h postdose and eliminated with a mean *T*_1/2_ value of 15 to 25 h postdose across all subjects (Fig. 1). M-I exhibited a similar mean plasma concentration-time profile to the parent TAK-063. No substantial differences for either analyte were observed between Japanese and non-Japanese subjects (Online Resource 5).

**Fig. 2:**
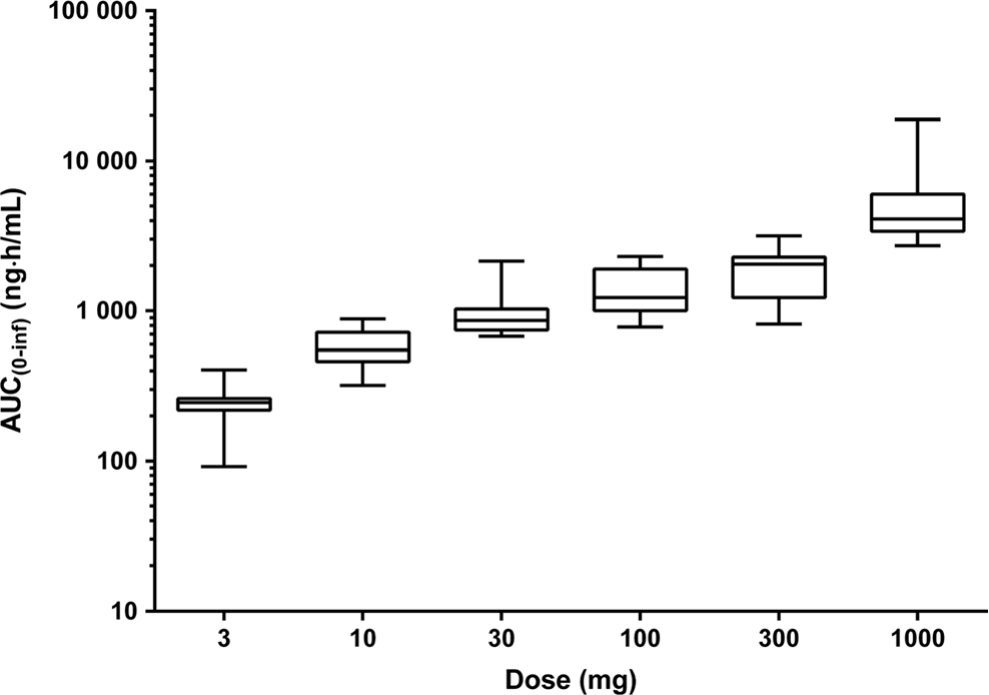
Box plots^a^ of TAK-063 exposure under fasting conditions across all subjects by dosing group. ^a^ *Middle lines* of boxes represent median values; *top and bottom borders* show lower and upper quartiles; *whiskers* represent minima and maxima

The plasma and urine PK parameters of TAK-063 and M-I under fasting conditions are presented in Table 3. Exposure to TAK-063 was less than dose-proportional with AUC values increasing up to 26-fold in response to the 333-fold increase in TAK-063 dose investigated in this study (Fig. 2). The intersubject variability of TAK-063 *C*_max_ and *AUC*_(0-inf)_ values was generally moderate across doses (18–81 % coefficient of variation). Clearance of TAK-063 (CL/F) increased with dose whereas renal elimination was negligible (Table 3). Similar PK properties were observed for TAK-063 M-I (M-I to parent *C*_max_ and AUC ratios: 0.82–0.97 and 0.78–1.04). PK parameters for Japanese and non-Japanese subjects are reported in Online Resources 3 and 4. For both TAK-063 and M-I, no substantial differences in pharmacokinetics were observed between Japanese and non-Japanese subjects.

**Table 3:** Plasma and urine PK parameters for TAK-063 and M-I under fasting conditions across all subjects by dosing group

	Arithmetic mean (% CV)											
	3 mg (*n* = 11)		10 mg (*n* = 11)		30 mg (*n* = 11)		100 mg (*n* = 11)		300 mg (*n* = 11)		1000 mg (*n* = 11)	
Parameter (unit)	TAK-063	M-I	TAK-063	M-I	TAK-063	M-I	TAK-063	M-I	TAK-063	M-I	TAK-063	M-I
Plasma												
*C*_max_ (ng/mL)	11.8 (51)	10.8 (47)	26.7 (34)	22.4 (32)	46.9 (61)	35.8 (46)	80.5 (48)	77.5 (57)	100.3 (18)	92.6 (21)	245.8 (34)	231.8 (45)
*T*_max_ (h)^a^	4.0 (2.0, 24.0)	4.0 (1.5, 24.0)	4.0 (2.0, 16.0)	4.0 (3.0, 16.0)	4.0 (3.0, 16.0)	4.0 (3.0, 6.0)	3.0 (1.5, 6.0)	3.0 (1.5, 6.0)	4.0 (1.9, 8.0)	4.0 (2.0, 8.0)	4.0 (2.0, 12.0)	4.0 (3.0, 12.0)
*AUC*_(0-inf)_ (ng·h/mL)	244.9 (32)	232.0 (48)^b^	576.7 (29)	509.0 (22)	1003.6 (42)	799.4 (48)	1406.8 (38)^c^	1487.0 (53)^c^	1900.8 (39)^d^	1891.0 (60) ^d^	5788.1 (81)	6290.5 (111)
*T*_1/2_ (h)	20.4 (48)	20.3 (52)^b^	19.9 (18)	21.4 (24)	20.4 (30)	20.1 (34)	24.6 (37)^c^	23.5 (32)^c^	20.2 (26)^d^	19.2 (37)^d^	14.6 (43)	15.4 (36)
CL/F (L/h)	14.0 (48)	15.6 (44)	18.7 (30)	20.7 (26)	33.1 (27)	45.1 (42)	80.3 (35)^c^	80.5 (39)	185.9 (48)^d^	213.1 (63)	232.0 (42)	253.8 (53)
V_z_/F (L)	407.0 (64)	414.6 (51)	529.5 (29)	651.3 (47)	954.8 (33)	1237.7 (37)	2802.2 (46)^c^	2619.1 (41)	5570.7 (57)^d^	5688.5 (59)	5277.2 (81)	5544.9 (68)
Urine												
CLr (mL/h)	0.0 (NA)	1.4 (332)	0.0 (NA)	4.9 (94)	2.4 (123)	14.8 (93)	5.9 (78)	16.7 (46)	6.1 (63)	15.6 (51)	6.8 (56)	17.0 (48)

*CL/F* oral clearance; *CLr* renal clearance; *CV* coefficient of variation; *M-I* metabolite; *NA* not applicable; *V*_*z*_ */F* volume of distribution

^a^ *T*_max_ is presented at the median (minimum, maximum)

^b^ *n* = 10, terminal phase of the PK profile could not be characterized in some subjects

^c^ *n* = 9, terminal phase of the PK profile could not be characterized in some subjects

^d^ *n* = 8, terminal phase of the PK profile could not be characterized in some subjects

An evaluation of the effects of food on absorption and oral bioavailability of TAK-063 following a 100-mg dose found that TAK-063 was absorbed more slowly when coadministered with a standard meal, (median *T*_max_ = 6 h, compared with 3 to 4 h under fasting condition), and oral bioavailability of TAK-063 was approximately 2-fold greater than under fasting conditions (Table 4). Terminal elimination, fraction of dose excreted, and renal clearance were not substantially altered under fed conditions. M-I showed similar PK properties to TAK-063 in the fasting and fed arms, with M-I to parent ratios of 0.97 and 1.00; 0.99 and 0.98; and 1.04 and 1.00 for *C*_max_, *AUC*_(0-tlqc)_, and *AUC*_(0-inf)_, respectively. No observed differences were apparent between Japanese and non-Japanese subjects in either fasting or fed condition (data not shown).

**Table 4:** PK parameters for food-effect arm following oral administration of 100 mg TAK-063 in Japanese and non-Japanese subjects

	TAK-063		M-I	
	Estimate	90 % CI	Estimate	90 % CI
*C*_max_ (ng/mL)	1.86	1.54, 2.25	1.95	1.63, 2.33
*AUC*_(0-inf)_ (ng·h/mL)	1.78	1.52, 2.09	1.75	1.44, 2.12

*CI* confidence interval; *M-I* metabolite

### Effects of TAK-063 on cognition

An overall dose effect of TAK-063 relative to placebo was observed at 2 and 6 h postdose (Online Resource 6) on the domains of cognitive flexibility, executive functioning, processing speed, psychomotor speed, and visual memory. In addition, statistically significant effects were noted at the 2-h timepoint in composite memory, reaction time, and verbal memory; the effects in these domains were not significant at the 6-h timepoint. A decrease from baseline in domain score in these cognitive domains, indicative of impairment, was generally observed following TAK-063 administration relative to the changes observed after placebo administration (Online Resource 6). No statistically significant differences were observed between Japanese and non-Japanese subjects for any of the neurocognitive domains tested (data not shown).

## Discussion

In this single-ascending-dose study, the PK, PD, safety, and tolerability profiles of TAK-063 in healthy subjects were evaluated under fasting and fed conditions. The results show that TAK-063 is safe and well tolerated following administration of a single dose in Japanese and non-Japanese volunteers. Specifically, treatment with a single dose of TAK-063 was not associated with hyperprolactinemia, hyperglycemia, or other metabolic disturbances that are often observed with current antipsychotics (Henderson et al. 2005; Lieberman et al. 2005; Miyamoto et al. 2005). Somnolence was the most common TEAE associated with TAK-063 treatment. Across all doses, the incidence of EPS-like AEs was negligible, occurring in only one Japanese patient at 30 mg (mild muscle tightness); it resolved without intervention. This may suggest that single doses of TAK-063 may have a relatively low propensity for EPS. No adverse events were considered dose-limiting, and a maximum tolerated dose was not defined.

TAK-063 and M-I exposure increased in a dose-dependent manner. The nonlinear PK of TAK-063 may be due to limitations in drug absorption and oral bioavailability; the low aqueous solubility of TAK-063 is consistent with preclinical toxicology studies in which the drug was excreted in the feces, suggesting that the test article was not absorbed. Drug absorption and oral bioavailability can be increased through coadministration with food, and TAK-063 treatment under fed conditions exhibited slower absorption and increased oral bioavailability. These results are consistent with previous studies in animal models assessing TAK-063’s solubility in the fed state.

The analyses of cognition data suggest that single doses of TAK-063 ≥ 100 mg cause impairments in cognitive flexibility, executive functioning, processing and psychomotor speed, and visual memory. While the small sample size limits the interpretation of the cognition results, higher incidences and severity of somnolence were also reported at higher doses of TAK-063 and these effects may be similar to the effects on cognition of other sedative or hypnotic agents (Miyata et al. 2015; Stranks and Crowe 2014). Together, these data will inform subsequent analyses of TAK-063 studies in subjects with schizophrenia.

Selective PDE10A inhibitors represent a potentially new strategy in the treatment of schizophrenia, with preliminary evidence supporting a favorable safety and tolerability profile, and preclinical studies suggest PDE-10A inhibitors may have the potential for efficacy across multiple symptom domains (Grauer et al. 2009; Kehler and Nielsen 2011; Suzuki et al. 2015). The data reported in this study demonstrate the safety and tolerability of TAK-063 following a single dose of up to 1000 mg and support once-daily dosing. Taken together, these preliminary findings are encouraging and support the need for additional studies of TAK-063 in patients with schizophrenia. A clinical trial to characterize the PK and PD profiles of multiple rising doses of TAK-063 in patients with schizophrenia has recently been completed (NCT01879722). A phase 2 study of the efficacy and safety of TAK-063 for the treatment of acute exacerbations of the symptoms of schizophrenia is currently being conducted (NCT02477020).

## Electronic supplementary material

ESM 1 (PDF 338 kb)
