# Supplementary material for: A phase 1 study of the safety, tolerability, pharmacokinetics, and pharmacodynamics of TAK-063, a selective PDE10A inhibitor
Source: Psychopharmacology (Berl). 2016 Aug 30;233(21):3787–95. doi: 10.1007/s00213-016-4412-9 (PMC5063900; doi:10.1007/s00213-016-4412-9)
Supplement: Supplementary file 1 — (PDF 338 kb) [file 213_2016_4412_MOESM1_ESM.pdf]

A Phase 1 Study of the Safety, Tolerability, Pharmacokinetics, and Pharmacodynamics of TAK-063, a Selective PDE10A Inhibitor

Max Tsai[1], Lambros Chrones[1], Jinhui Xie[1], Hakop Gevorkyan[2], and Thomas A. Macek[1*]

[1]Takeda Development Center Americas, Inc., Deerfield, IL, USA; [2]California Clinical Trials Medical Group, Glendale, CA, USA

*Corresponding author at: One Takeda Parkway, Deerfield, IL 60015. Tel: +1 224-554-2803; Fax: 224-554-3637

Email address: tom.macek@takeda.com (T. Macek)

Online Resource 1

Treatment-emergent adverse events for Japanese subjects by dosing group

Adverse event, n (%)	Placebo (n = 6)	TAK-063 3 mg (n = 5)	TAK-063 10 mg (n = 5)	TAK-063 30 mg (n = 5)	TAK-063 100 mg (n = 5)	TAK-063 300 mg (n = 5)	TAK-063 1000 mg (n = 5)	TAK-063 Total (n = 30)
Somnolence	0	0	0	1 (20.0)	4 (80.0)	2 (40.0)	3 (60.0)	10 (33.3)
Orthostatic tachycardia	1 (16.7)	0	0	1 (20.0)	0	1 (20.0)	3 (60.0)	5 (16.7)
Orthostatic hypotension	1 (16.7)	0	0	0	1 (20.0)	1 (20.0)	2 (40.0)	4 (13.3)
Vomiting	0	0	0	0	1(20.0)	0	1 (20.0)	2 (6.7)
Nausea	0	0	0	0	0	0	1 (20.0)	1 (3.3)
Dizziness	0	0	0	0	0	0	1 (20.0)	1 (3.3)
Headache	0	0	0	0	1 (20.0)	0	0	1 (3.3)
Epistaxis	0	0	0	1 (20.0)	0	0	0	1 (3.3)
Muscle tightness	0	0	0	1 (20.0)	0	0	0	1 (3.3)

Online Resource 2

Treatment-emergent adverse events for non-Japanese subjects by dosing group

Adverse event, n (%)	Placebo (n = 12)	TAK-063 3 mg (n = 6)	TAK-063 10 mg (n = 6)	TAK-063 30 mg (n = 6)	TAK-063 100 mg (n = 6)	TAK-063 300 mg (n = 6)	TAK-063 1000 mg (n = 6)	TAK-063 Total (n = 36)
Somnolence	0	0	0	3 (50.0)	3 (50.0)	3 (50.0)	3 (50.0)	12 (33.3)
Orthostatic tachycardia	0	2 (33.3)	0	0	2 (33.3)	0	4 (66.7)	8 (22.2)
Orthostatic hypotension	2 (16.7)	0	0	0	0	0	3 (50.0)	3 (8.3)
Vomiting	0	0	0	0	0	1 (16.7)	0	1 (2.8)
Nausea	0	0	0	2 (33.3)	0	0	0	2 (5.6)
Dizziness	0	0	0	1 (16.7)	0	1 (16.7)	0	2 (5.6)
Dysarthria	0	0	0	0	0	0	1 (16.7)	1 (2.8)
Headache	2 (16.7)	0	0	0	0	1 (16.7)	0	1 (2.8)
Anxiety	0	0	0	0	0	0	1 (16.7)	1 (2.8)
Hypotension	0	0	0	0	1 (16.7)	0	0	1 (2.8)
Blurred vision	0	0	0	0	0	0	1 (16.7)	1 (2.8)

Online Resource 3

PK parameters for Japanese subjects by dosing group

	Arithmetic mean (% CV)											
	3 mg (n = 5)		10 mg (n = 5)		30 mg (n = 5)		100 mg (n = 5)		300 mg (n = 5)		1000 mg (n = 5)	
**Parameter (unit)**	**TAK-063**	**M-I**	**TAK-063**	**M-I**	**TAK-063**	**M-I**	**TAK-063**	**M-I**	**TAK-063**	**M-I**	**TAK-063**	**M-I**
**Plasma**												
C_{max} (ng/mL)	16.1 (42)	13.3 (45)	30.2 (32)	20.8 (39)	55.7 (67)	36.9 (55)	89.9 (41)	78.1 (33)	98.2 (25)	84.1 (17)	264.0 (28)	234.8 (45)
T_{max} (h)[a]	4.0 (2.0, 4.0)	4.0 (2.0, 4.0)	4.0 (2.0, 16.0)	4.0 (3.0, 16.0)	4.0 (3.0, 6.0)	4.0 (4.0, 6.0)	3.0 (3.0, 4.1)	3.0 (2.0, 4.1)	4.0 (2.0, 8.0)	4.0 (2.0, 4.0)	4.0 (2.0, 4.0)	3.0 (3.0, 8.0)
$AUC_{(0-inf)}$ (ng·h/mL)	250.3 (22)	206.4 (26)	659.2 (23)	488.7 (27)	1027.3 (61)	674.0 (65)	1459.1 (37)[b]	1364.9 (27)[b]	1636.0 (38)[b]	1410.8 (46)[b]	4931.4 (46)	4343.1 (47)
$T_{1/2}$ (h)	15.9 (67)	16.5 (80)	18.2 (17)	20.1 (37)	17.8 (42)	15.4 (43)	20.7 (35)[b]	20.5 (33)[b]	20.3 (37)[b]	19.5 (52)[b]	11.7 (26)	11.9 (14)
CL/F (L/h)	12.5 (24)	15.6 (32)	15.8 (22)	21.9 (31)	35.2 (35)	55.5 (41)	75.8 (35)[b]	77.4 (27)	212.8 (50)[b]	266.4 (62)	233.8 (39)	283.5 (55)
V_z/F (L)	269.5 (49)	335.1 (57)	412.2 (23)	679.2 (69)	889.4 (50)	1226.9 (47)	2288.5 (59)[b]	2272.3 (44)	6409.8 (60)[b]	6862.4 (59)	3953.4 (44)	4763.4 (46)
**Urine**												
CL_r (mL/h)	0.0 (NA)	3.0 (224)	0.0 (NA)	7.8 (47)	4.0 (85)	22.0 (73)	7.9 (67)	17.4 (50)	5.9 (71)	16.6 (53)	6.8 (57)	18.9 (47)

Abbreviations: CL/F, oral clearance; CL_r, renal clearance; CV, coefficient of variation; M-I, metabolite; NA, not applicable; V_z/F, volume of distribution

[a] T_{max} is presented at the median (minimum, maximum)

[b] n = 4, terminal phase of the PK profile could not be characterized in some subjects

Online Resource 4
PK parameters for non-Japanese subjects by dosing group

Parameter (unit)	Arithmetic mean (% CV)											
	3 mg (n = 6)		10 mg (n = 6)		30 mg (n = 6)		100 mg (n = 6)		300 mg (n = 6)		1000 mg (n = 6)	
	TAK-063	M-I	TAK-063	M-I	TAK-063	M-I	TAK-063	M-I	TAK-063	M-I	TAK-063	M-I
**Plasma**												
C_{max} (ng/mL)	8.2 (21)	8.8 (39)	23.8 (35)	23.8 (28)	39.5 (47)	34.8 (41)	72.7 (57)	76.9 (75)	102.1 (13)	99.7 (21)	230.7 (41)	229.3 (49)
T_{max} (h)[a]	5.0 (2.0, 24.0)	3.5 (1.5, 24.0)	5.0 (2.0, 8.0)	5.0 (3.0, 6.0)	5.0 (3.0, 16.0)	3.5 (3.0, 6.0)	3.5 (1.5, 6.0)	4.0 (1.5, 6.0)	3.5 (1.9, 4.0)	3.5 (2.9, 8.0)	4.0 (3.0, 12.0)	5.0 (3.0, 12.0)
$AUC_{(0\text{-}inf)}$ (ng·h/mL)	240.4 (42)	257.6 (59)[c]	507.9 (30)	526.0 (20)	983.9 (20)	903.9 (38)	1364.9 (44)[c]	1584.6 (66)[c]	2165.7 (39)[b]	2371.2 (59)[b]	6502.0 (95)	7913.4 (118)
$T_{1/2}$ (h)	24.2 (34)	24.0 (29)[c]	21.3 (17)	22.4 (10)	22.6 (18)	24.1 (17)	27.8 (36)[c]	25.9 (31)[c]	20.0 (15)[b]	18.9 (18)[b]	17.0 (44)	18.2 (34)
CL/F (L/h)	15.2 (58)	15.5 (58)	21.2 (29)	19.7 (21)	31.4 (18)	36.4 (29)	84.0 (39)[c]	83.0 (49)	159.0 (47)[b]	159.7 (52)	230.6 (47)	228.9 (53)
V_z/F (L)	521.6 (57)	494.0 (44)	627.3 (19)	628.1 (13)	1009.2 (19)	1246.8 (31)	3213.2 (38)[c]	2896.6 (41)	4731.5 (54)[b]	4514.6 (57)	6380.3 (87)	6196.3 (78)
**Urine**												
CL_r (mL/h)	0.0 (NA)	0.0 (NA)	0.0 (NA)	2.5 (160)	1.1 (179)	8.8 (100)	4.2 (84)	16.1 (47)	6.2 (63)	14.8 (53)	6.8 (61)	15.4 (51)

CV, coefficient of variation; M-I, metabolite; NA, not applicable
[a] T_{max} is presented at the median (minimum, maximum)
[b] n = 4, terminal phase of the PK profile could not be characterized in some subjects
[c] n = 5, terminal phase of the PK profile could not be characterized in some subjects

Online Resource 5

TAK-063 and M-I concentration time profiles across all subjects by dosing group, stratified by race

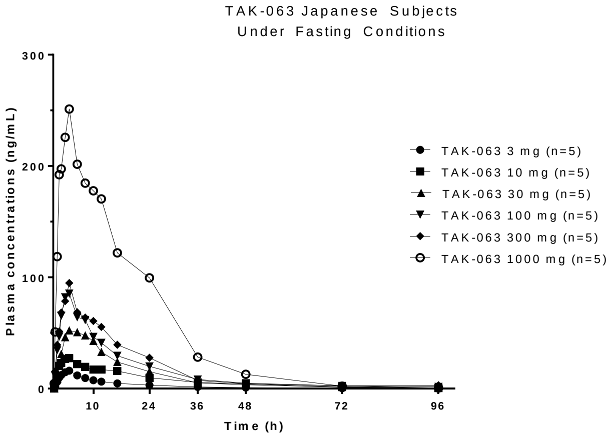

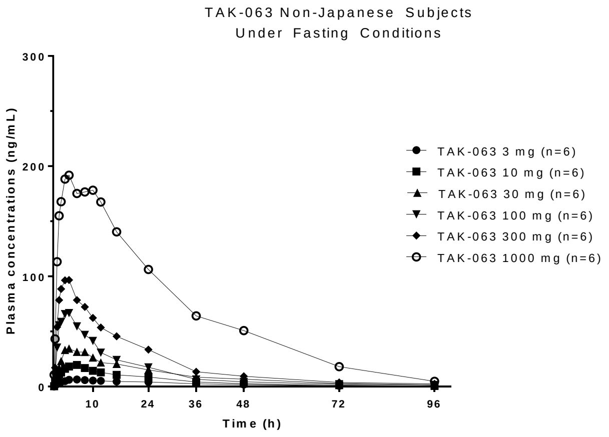

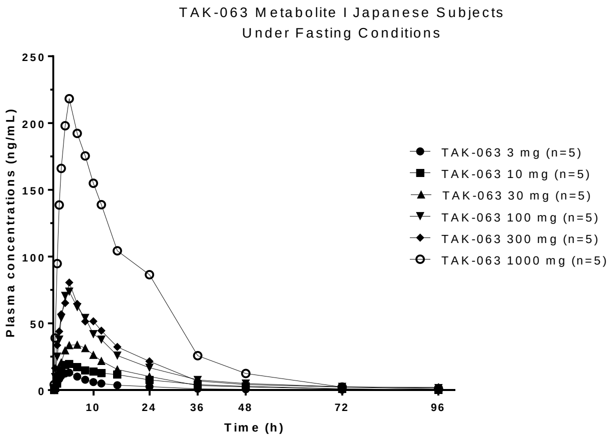

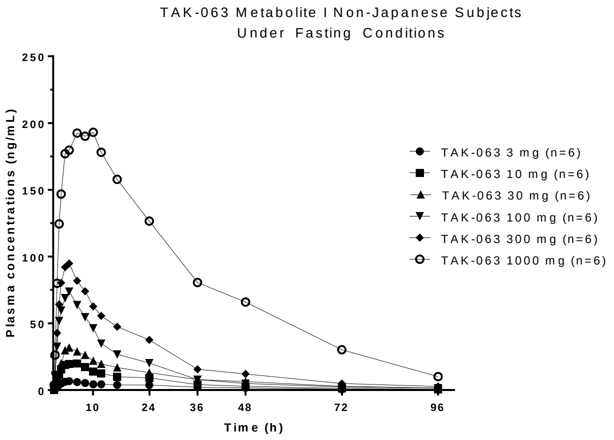

Online Resource 6

Statistical analysis of standardized domain scores from neurocognitive tests under the fasting condition

	Cognitive flexibility		Composite memory		Executive functioning		Processing speed		Psychomotor speed		Reaction time		Reasoning		Sustained attention		Verbal memory		Visual memory		Working memory	
	Difference[a]		Difference[a]		Difference[a]		Difference[a]		Difference[a]		Difference[a]		Difference[a]		Difference[a]		Difference[a]		Difference[a]		Difference[a]	
	**2 h**	**6 h**	**2 h**	**6 h**	**2 h**	**6 h**	**2 h**	**6 h**	**2 h**	**6 h**	**2 h**	**6 h**	**2 h**	**6 h**	**2 h**	**6 h**	**2 h**	**6 h**	**2 h**	**6 h**	**2 h**	**6 h**
3 mg	1.3	2.9	-7.9	-1.4	0.4	2.9	2.2	2.5	-1.5	-1.8	4.1	1.5	5.7	6.9	4.6	15.0	0.9	-4.8	-10.3	4.5	5.3	9.9
10 mg	-3.1	-10.0	-5.2	-1.9	-2.7	-10.0	0.7	0.2	-8.5	-9.6	-2.3	-5.6	10.8	2.2	0.7	3.6	-4.0	2.9	-3.3	-4.4	1.1	2.3
30 mg	0.2	-1.9	2.8	7.5	0.5	-1.2	4.3	2.2	-1.7	-3.7	3.1	-2.8	6.4	-0.6	10.3	2.5	12.4	3.8	-5.2	8.3	6.5	-2.2
100 mg	-20.2	-5.6	-24.1	-13.5	-17.8	-2.2	-6.1	-4.7	-15.2	-11.4	-15.6	-3.4	4.6	2.0	-7.0	-0.1	-24.0	-12.0	-15.4	-8.3	-3.8	-6.2
300 mg	-30.9	-28.4	-26.9	-19.6	-29.0	-26.4	-17.6	-1.5	-18.0	-11.1	-10.3	-4.1	0.5	-2.0	-9.0	-8.1	-26.7	-17.3	-17.2	-14.0	-3.1	-1.4
1000 mg	-19.6	-15.6	-16.6	-8.6	-16.6	-14.1	-17.9	-19.7	-18.7	-22.7	-7.1	-16.5	-0.5	-0.1	-5.0	0.7	-10.0	-5.8	-14.5	-6.0	-2.5	-2.7
P value for overall dose effect[b]	0.013	0.010	<0.001	0.051	0.032	0.015	<0.001	0.001	<0.001	<0.001	0.007	0.079	0.450	0.797	0.311	0.189	<0.001	0.314	0.049	0.038	0.527	0.135

ANCOVA; analysis of covariance

[a]TAK-063 least square means – placebo least square means on change from baseline; decreased score is indicative of impairment

[b]*P* values based on the comparison between each TAK-063 dose level and placebo within the frame of the ANCOVA model
